# Otis Clapp & Son’s Visiting List

**Published:** 1887-12

**Authors:** 


### Otis Clapp & Son's Visiting List and Prescription Record. Boston and Providence: Otis Clapp & Son.

As the new year approaches, physicians are beginning to look around for suitable visiting lists in which to record the work of the coming year. To most of them who practice Homœopathy, the list issued by Otis Clapp & Son is well known, and needs no recommendation from us. There may be some, however, who are not familiar with it. For the benefit of such, we may say that the one here noticed possesses three special advantages: First, it is perpetual, not being confined to any one year; secondly, the columns are so arranged that the remedy may be noted; thirdly, in the front of the book is a list of the principal remedies, each one numbered, so that to note the remedy given it is only necessary to set down its number, and thus the difficulty of crowding a long name into a small space, and yet making it legible, is obviated. Like other lists, it has the usual tables of antidotes to poisons, etc. W. M. J.

